# Supplementary material for: Quantum mechanics and the covariance of physical laws in quantum reference frames
Source: Nat Commun. 2019 Jan 30;10:494. doi: 10.1038/s41467-018-08155-0 (PMC6353997; doi:10.1038/s41467-018-08155-0)
Supplement: Supplementary file 1 — Supplementary Information [file 41467_2018_8155_MOESM1_ESM.pdf]

Supplementary Information

Quantum mechanics and the covariance of physical laws in quantum reference frames

Giacomini et al.

SUPPLEMENTARY NOTE 1: COORDINATE TRANSFORMATIONS BETWEEN REFERENCE FRAMES

We briefly review the covariance of the Schrödinger equation for a non-relativistic free particle under Galilean transformations between classical reference frames [1]. These reference frames are abstract notions and are not considered to be physical degrees of freedom with their own dynamics. Among all possible transformations between two reference frames, an important role is played by the extended Galilean transformations, which take the form $x' = x - X(t)$, $t' = t$ (see Refs. [2, 3]). This tranformations include the Galilean symmetries such as translations ($X(t) = X_0$) and boosts ($X(t) = vt$, where v is the velocity of the new reference frame with respect to the old one), and also the transformation to an accelerated reference frame ($X(t) = \frac{1}{2}at^2$, where a is the acceleration of the reference frame), which plays an important role in statements of the weak equivalence principle. Consider the Schrödinger equation of a particle moving freely in one dimension from the perspective of an inertial reference system S_0

$$\mathrm{i}\hbar\frac{\partial\psi(x,t)}{\partial t} = -\frac{\hbar^2}{2m}\frac{\partial^2\psi(x,t)}{\partial x^2}. \tag{1}$$

The coordinates (x', t') of the particle with respect to a distinct reference system S_1, whose position at time t relative to S_0 is $X(t)$, are obtained through $x' = x - X(t)$ and $t' = t$, where (x, t) are the space-time coordinates in S_0. Using $\frac{\partial}{\partial x} = \frac{\partial}{\partial x'}$ and $\frac{\partial}{\partial t} = \frac{\partial}{\partial t'} - \dot{X}\frac{\partial}{\partial x'}$ one obtains the Schrödinger equation in the reference frame S_1

$$\mathrm{i}\hbar\frac{\partial\psi'(x',t')}{\partial t'} = (-\frac{\hbar^2}{2m}\frac{\partial^2}{\partial x'^2} + \mathrm{i}\hbar\dot{X}\frac{\partial}{\partial x'})\psi'(x',t'), \tag{2}$$

where $\psi'(x', t') = \psi(x - X(t), t)$ and we take into account that time is absolute in non-relativistic quantum mechanics.

In the case of spatial translations, where $X(t) = X_0$, it is immediate to verify that the Schrödinger equation is invariant under this transformation. An equivalent way of describing this transformation is via the translation operator $\hat{T}_{X_0} = e^{\frac{\mathrm{i}}{\hbar}X_0\hat{P}}$, which shifts the position of the system by X_0 in the x direction. For a state $|\psi\rangle = \int dx\psi(x)|x\rangle$, the action of the translation operator is $\hat{T}_{X_0}|\psi\rangle = \int dx\,\psi(x + X_0)|x\rangle$. Translations are symmetries of the N-particle Hamiltonian $\hat{H} = \sum_{i=1}^{N}\frac{\hat{p}_i}{2m_i} + \sum_{i\leq j}V_{ij}(\hat{x}_i - \hat{x}_j)$, where V_{ij} is the potential of the pairwise interaction between particles i and j, since they leave the Hamiltonian invariant.

If $\dot{X} = v$ is constant, the extended Galilean transformation implements the Galilean boost. The Hamiltonian is invariant under the transformation provided that the state changes according to $\tilde{\psi}(x', t') = e^{-\frac{\mathrm{i}}{\hbar}(mvx' + \frac{m}{2}v^2t')}\psi'(x', t')$. This transformation can also be found by applying the unitary representation of the boost directly to the initial state

$$|\tilde{\psi}\rangle = e^{\frac{\mathrm{i}}{\hbar}v\hat{G}}|\psi\rangle, \tag{3}$$

where $\hat{G} = t\hat{p} - m\hat{x}$ is the generator of Galilean boosts.

The last transformation we consider is the transformation to an accelerated reference frame, i.e. $X(t) = \frac{1}{2}at^2$. In this case, if the quantum state in the accelerated reference frame S_1 acquires the additional phase

$$\tilde{\psi}(x', t') = e^{-\frac{\mathrm{i}}{\hbar}(m\dot{X}x' + \frac{m}{2}\int_0^{t'} ds \dot{X}^2(s))}\psi'(x', t'), \tag{4}$$

the state $\tilde{\psi}(x', t')$ satisfies the Schrödinger equation

$$\mathrm{i}\hbar\frac{\partial\tilde{\psi}(x', t')}{\partial t'} = (-\frac{\hbar^2}{2m}\frac{\partial^2}{\partial x'^2} + m\ddot{X}x')\tilde{\psi}(x', t'). \tag{5}$$

This result shows that moving to an accelerated frame is equivalent to placing the quantum system in a linear gravitational field with acceleration $\ddot{X} = a$. In this sense, it provides a way to test the weak equivalence principle (WEP), according to which physics is the same from the point of view of an observer moving with uniform acceleration a or from the point of view of the same observer standing on the surface of the Earth, where the gravitational costant g is equal to a, but directed in the opposite direction [4].

It is convenient to look at the symmetries of the Hamiltonian as gauge symmetries. Quantum mechanics can be cast as a gauge theory [5]. Here, time is the basis manifold, and the Hamiltonian $\hat{H}$ is the gauge field. The Schrödinger equation takes the form of a vanishing covariant derivative with respect to time $\mathcal{D}|\psi\rangle = \left(\partial_t + \frac{\mathrm{i}}{\hbar}\hat{H}\right)|\psi\rangle = 0$. The gauge symmetry is a non-Abelian, local symmetry $\hat{U}(t)$, which acts on the vector field (i.e. the quantum state) as $|\psi'\rangle = \hat{U}(t)|\psi\rangle$. It is straightforward to see that the covariant derivative satisfies the condition $\hat{U}(t)\mathcal{D}|\psi\rangle = \mathcal{D}'\hat{U}(t)|\psi\rangle = 0$ if $\mathcal{D}' = \partial_t + \frac{\mathrm{i}}{\hbar}\hat{H}'$ and the Hamiltonian transforms like a connection, i.e. $\hat{H}' = \hat{U}(t)\hat{H}\hat{U}^{-1}(t) + \mathrm{i}\hbar(\partial_t\hat{U}(t))\hat{U}^{-1}(t)$. A theory which implements these conditions possesses a gauge symmetry. It is easy to see that the extended Galilean transformations introduced previously in the paragraph are examples of such gauge transformations. In particular, we say that translations and boosts are symmetries of the free-particle Hamiltonian $\hat{H} = \frac{\hat{p}^2}{2m}$, because both gauge transformations $e^{\frac{\mathrm{i}}{\hbar}X_0\hat{p}}$, corresponding to translations, and $e^{\frac{\mathrm{i}}{\hbar}v\hat{G}}$, corresponding to Galilean boosts, yield $\hat{H}' = \hat{H}$.

SUPPLEMENTARY NOTE 2: TRANSITIVITY OF THE REFERENCE FRAME TRANSFORMATION

We show that the transformation $\hat{S}_{\mathrm{x}} = \hat{\mathcal{P}}_{\mathrm{AC}}e^{\frac{\mathrm{i}}{\hbar}\hat{x}_{\mathrm{A}}\hat{p}_{\mathrm{B}}}$ satisfies the transitive property. The same procedure can be followed to show the same for the transformations $\hat{S}_{\mathrm{T}}$ (in Methods-Translations between quantum reference frames) and $\hat{S}_{\mathrm{b}}$ (in Methods-Boosts between quantum reference frames).

Let us consider a completely general state of A and B $|\Psi\rangle_{\mathrm{AB}}^{(\mathrm{C})}$, as described in the reference frame C. For simplicity, we assume it to be a pure state, but the argument can be extended by linearity to

mixed states. We want to show that changing reference frame from C to B and then from B to A gives the same result as changing it from C to A, i.e. $\hat{S}_{\mathrm{x}}^{(\mathrm{B}\to\mathrm{A})}\hat{S}_{\mathrm{x}}^{(\mathrm{C}\to\mathrm{B})} = \hat{S}_{\mathrm{x}}^{(\mathrm{C}\to\mathrm{A})}$. For brevity of notation, we only consider the three systems A, B, and C, but it is straightforward to verify that the same argument holds for an arbitrary number of systems involved in the transformation. By expanding the initial state in position basis, $|\Psi\rangle_{\mathrm{AB}}^{(\mathrm{C})} = \int dx_{\mathrm{A}}dx_{\mathrm{B}}\Psi(x_{\mathrm{A}}, x_{\mathrm{B}})\,|x_{\mathrm{A}}\rangle_{\mathrm{A}}\,|x_{\mathrm{B}}\rangle_{\mathrm{B}}$ and applying the transformation $\hat{S}_{\mathrm{x}}^{(\mathrm{C}\to\mathrm{B})}$ to the reference frame B, we obtain

$$\hat{S}_{\mathrm{x}}^{(\mathrm{C}\to\mathrm{B})}\,|\Psi\rangle_{\mathrm{AB}}^{(\mathrm{C})} = \int dk_{\mathrm{A}}dk_{\mathrm{C}}\Psi(k_{\mathrm{A}} - k_{\mathrm{C}}, -k_{\mathrm{C}})\,|k_{\mathrm{A}}\rangle_{\mathrm{A}}\,|k_{\mathrm{C}}\rangle_{\mathrm{C}}\,. \tag{6}$$

We now apply the transformation $\hat{S}_{\mathrm{x}}^{(\mathrm{B}\to\mathrm{A})}$ to the transformed state to go to reference frame A

$$\hat{S}_{\mathrm{x}}^{(\mathrm{B}\to\mathrm{A})}\hat{S}_{\mathrm{x}}^{(\mathrm{C}\to\mathrm{B})}\,|\Psi\rangle_{\mathrm{AB}}^{(\mathrm{C})} = \int dq_{\mathrm{B}}dq_{\mathrm{C}}\Psi(-q_{\mathrm{C}}, q_{\mathrm{B}} - q_{\mathrm{C}})\,|q_{\mathrm{B}}\rangle_{\mathrm{B}}\,|q_{\mathrm{C}}\rangle_{\mathrm{C}}\,, \tag{7}$$

which is the same result we get when we apply $\hat{S}_{\mathrm{x}}^{(\mathrm{C}\to\mathrm{B})}$ directly to the initial state. In a similar way it can be seen that $\hat{S}_{\mathrm{x}}^{(\mathrm{A}\to\mathrm{C})}\hat{S}_{\mathrm{x}}^{(\mathrm{C}\to\mathrm{A})}\,|\Psi\rangle_{\mathrm{AB}}^{(\mathrm{C})} = |\Psi\rangle_{\mathrm{AB}}^{(\mathrm{C})}$.

We can now generalise the argument to show that, if we consider $N+1$ systems $\mathrm{A}_0, \ldots, \mathrm{A}_N$ and we wish to tranform from the QRF A_0 to the QRF A_N, it is equivalent to apply the transformation $\hat{S}_{\mathrm{x}}^{(\mathrm{A}_0\to\mathrm{A}_N)}$ to the state in the initial QRF A_0 or to apply a chain of transformations $\hat{S}_{\mathrm{x}}^{(\mathrm{A}_{\mathrm{N}-1}\to\mathrm{A}_{\mathrm{N}})}\cdots\hat{S}_{\mathrm{x}}^{(\mathrm{A}_1\to\mathrm{A}_2)}\hat{S}_{\mathrm{x}}^{(\mathrm{A}_0\to\mathrm{A}_1)}$. This is easily proved by observing that the transitive property applies to each composition of two transformations $\hat{S}_{\mathrm{x}}^{(\mathrm{A}_{\mathrm{i}}\to\mathrm{A}_{\mathrm{i}+1})}\hat{S}_{\mathrm{x}}^{(\mathrm{A}_{\mathrm{i}-1}\to\mathrm{A}_{\mathrm{i}})}$, $1 \leq i \leq N-1$. Therefore, it also holds for the full transformation $\hat{S}_{\mathrm{x}}^{(\mathrm{A}_{\mathrm{N}-1}\to\mathrm{A}_{\mathrm{N}})}\cdots\hat{S}_{\mathrm{x}}^{(\mathrm{A}_1\to\mathrm{A}_2)}\hat{S}_{\mathrm{x}}^{(\mathrm{A}_0\to\mathrm{A}_1)}$. Please note that it is indifferent to the argument whether there is a system B additionally to the QRFs $\{\mathrm{A}_i\}_{i=0}^{N}$ or not.

SUPPLEMENTARY NOTE 3: CONSERVATION OF THE DYNAMICAL CONSERVED QUANTITIES UNDER QUANTUM REFERENCE FRAME TRANSFORMATION

Let us consider a unitary transformation from the reference frame C to A $\hat{S} : \mathcal{H}_{\mathrm{A}}^{(\mathrm{C})} \otimes \mathcal{H}_{\mathrm{B}}^{(\mathrm{C})} \to \mathcal{H}_{\mathrm{B}}^{(\mathrm{A})} \otimes \mathcal{H}_{\mathrm{C}}^{(\mathrm{A})}$, where the initial and final Hilbert spaces are isomorphic. In C, the dynamical evolution is described by the Hamiltonian $H_{\mathrm{AB}}^{(\mathrm{C})}$, and the quantum state in the Schrödinger picture is $\rho_{\mathrm{AB}}^{(\mathrm{C})}$. We say that an observable $\hat{C}^{(\mathrm{C})}$ is a dynamical conserved quantity if the condition $\frac{d}{dt}\mathrm{Tr}\left(\hat{C}^{(\mathrm{C})}\rho_{\mathrm{AB}}^{(\mathrm{C})}\right) = \mathrm{Tr}\left\{\frac{d\hat{C}^{(\mathrm{C})}}{dt} + \frac{\mathrm{i}}{\hbar}\left[H_{\mathrm{AB}}^{(\mathrm{C})}, \hat{C}^{(\mathrm{C})}\right]\right\}\rho_{\mathrm{AB}}^{(\mathrm{C})} = 0$ holds. It is then easy to see, by transforming with the reference frame transformation and using the cyclicity of the trace, that this condition is mapped, in the reference frame of A, to $\frac{d}{dt}\mathrm{Tr}\left(\hat{C}^{(\mathrm{A})}\rho_{\mathrm{BC}}^{(\mathrm{A})}\right) = \mathrm{Tr}\left\{\frac{d\hat{C}^{(\mathrm{A})}}{dt} + \frac{\mathrm{i}}{\hbar}\left[H_{\mathrm{BC}}^{(\mathrm{A})}, \hat{C}^{(\mathrm{A})}\right]\right\}\rho_{\mathrm{BC}}^{(\mathrm{A})} = 0$, where $H_{\mathrm{BC}}^{(\mathrm{A})}$ and $\rho_{\mathrm{BC}}^{(\mathrm{A})}$ are respectively the transformed Hamiltonian and quantum state in the frame of A, provided that $\hat{C}^{(\mathrm{A})} = \hat{S}\hat{C}^{(\mathrm{C})}\hat{S}^{\dagger}$. Therefore, if $\hat{C}^{(\mathrm{C})}$ is a conserved quantity in the reference frame of C, the transformed observable $\hat{C}^{(\mathrm{A})} = \hat{S}\hat{C}^{(\mathrm{C})}\hat{S}^{\dagger}$ is also a conserved quantity in the reference frame of A.

Let us now consider the set of all the N conserved quantities in the reference frame of C, i.e. $\left\{\hat{C}_i^{(\mathrm{C})}\right\}_{i=1,\ldots,N}$. Because the transformation $\hat{S}$ is unitary, in the reference frame of A there will also be N conserved quantities. In particular, all the transformed quantities $\hat{C}_i^{(\mathrm{A})} = \hat{S}\hat{C}_i^{(\mathrm{C})}\hat{S}^\dagger$, $i = 1, \ldots, N$, and their linear combinations $\sum_{i=1}^N \alpha_i \hat{C}_i^{(\mathrm{A})}$, with $\alpha_i \in \mathbb{R}$, are also conserved quantities in the reference frame of A.

If the transformation $\hat{S}$ is a symmetry for the quantum reference frame transformation, meaning that it casts the new Hamiltonian $H_{\mathrm{BC}}^{(\mathrm{A})}$ in the same functional form as the initial Hamiltonian $H_{\mathrm{AB}}^{(\mathrm{C})}$, but with the labels A and C swapped, then it must be that the new Hamiltonian has a set of N conserved quantities which have the same functional form of the conserved quantities in the reference frame A, but with the labels A and C swapped. Therefore, we can rearrange the set of the transformed observables $\hat{C}_i^{(\mathrm{A})}$ into a new set $\hat{C}_i^{\prime(\mathrm{A})} = \sum_{j=1}^N \gamma_i^j \hat{C}_j^{(\mathrm{A})}$, where $i = 1, \ldots, N$ and the vectors $\vec{\gamma}_i$ form a linearly independent set, such that the $\hat{C}_i^{\prime(\mathrm{A})}$ take the same functional form as in C, but with the labels C instead of A. Note that the linear combination is needed because the $\hat{S}$ transformation does not necessarily preserve the functional form of the conserved quantities. For example, see how momenta change in Methods-Translations between quantum reference frames and Methods-Boosts between quantum reference frames.

SUPPLEMENTARY NOTE 4: RELATIVE VELOCITY BETWEEN QUANTUM REFERENCE FRAMES

We consider the instantaneous transformation to the relative velocities between two QRFs

$$\hat{S}_{\mathrm{v}} = \hat{\mathcal{P}}_{\mathrm{AC}}^{(\mathrm{v})} \exp\left(-\frac{\mathrm{i}}{\hbar}\frac{m_{\mathrm{B}}}{m_{\mathrm{A}}}\hat{x}_{\mathrm{B}}\hat{p}_{\mathrm{A}}\right), \tag{8}$$

where the ‘generalised parity operator’ $\hat{\mathcal{P}}_{\mathrm{AC}}^{(\mathrm{v})} = \hat{\mathcal{P}}_{\mathrm{AC}} \exp\left(\frac{\mathrm{i}}{\hbar}\log\sqrt{\frac{m_{\mathrm{C}}}{m_{\mathrm{A}}}}(\hat{x}_{\mathrm{A}}\hat{p}_{\mathrm{A}} + \hat{p}_{\mathrm{A}}\hat{x}_{\mathrm{A}})\right)$ maps the velocity of A to the opposite of the velocity of C via the standard parity-swap operator $\hat{\mathcal{P}}_{\mathrm{AC}}$ and an operator scaling coordinates and momenta. Specifically, $\hat{\mathcal{P}}_{\mathrm{AC}}^{(\mathrm{v})}\hat{x}_{\mathrm{A}}\left(\hat{\mathcal{P}}_{\mathrm{AC}}^{(\mathrm{v})}\right)^\dagger = -\frac{m_{\mathrm{C}}}{m_{\mathrm{A}}}\hat{q}_{\mathrm{C}}$, $\hat{\mathcal{P}}_{\mathrm{AC}}^{(\mathrm{v})}\hat{p}_{\mathrm{A}}\left(\hat{\mathcal{P}}_{\mathrm{AC}}^{(\mathrm{v})}\right)^\dagger = -\frac{m_{\mathrm{A}}}{m_{\mathrm{C}}}\hat{\pi}_{\mathrm{C}}$. The transformation (8) corresponds to the boost transformation $\hat{S}_{\mathrm{b}}$ in Methods-Boosts between quantum reference frames for $t = 0$ and implements the following coordinate transformation

$$\begin{aligned} \hat{q}_{\mathrm{B}} &\mapsto \hat{x}_{\mathrm{B}}, & \hat{\pi}_{\mathrm{B}} &\mapsto \hat{p}_{\mathrm{B}} - \frac{m_{\mathrm{B}}}{m_{\mathrm{A}}}\hat{p}_{\mathrm{A}}, \\ \hat{q}_{\mathrm{C}} &\mapsto -\frac{1}{m_{\mathrm{C}}}\left(m_{\mathrm{A}}\hat{x}_{\mathrm{A}} + m_{\mathrm{B}}\hat{x}_{\mathrm{B}}\right), & \hat{\pi}_{\mathrm{C}} &\mapsto -\frac{m_{\mathrm{C}}}{m_{\mathrm{A}}}\hat{p}_{\mathrm{A}}, \end{aligned} \tag{9}$$

which corresponds to the transformation to the relative velocities when the initial Hamiltonian is quadratic in momenta. We find that the free-particle Hamiltonian is not invariant under this transformation. The Hamiltonian which is invariant, in the generalised sense expressed in the main text, is $\hat{H}_{\mathrm{AB}}^{(\mathrm{C})} = \frac{\hat{p}_{\mathrm{A}}^2}{2m_{\mathrm{A}}} + \frac{\hat{p}_{\mathrm{B}}^2}{2m_{\mathrm{B}}} - \frac{(\hat{p}_{\mathrm{A}}+\hat{p}_{\mathrm{B}})^2}{2M}$, with $M = m_{\mathrm{A}} + m_{\mathrm{B}} + m_{\mathrm{C}}$.

SUPPLEMENTARY NOTE 5: WEAK EQUIVALENCE PRINCIPLE FOR QUANTUM PARTICLES IN A GENERAL POTENTIAL

We show here how it is possible to relax the condition of having a piecewise linear potential to recover a generalised version of the weak equivalence principle, that is valid for general potentials acting for infinitesimal times. Let us consider the Hamiltonian in Methods-The weak equivalence principle in quantum reference frames, $\hat{H}^{(\mathrm{C})}_{\mathrm{AB}} = \frac{\hat{p}_{\mathrm{A}}^2}{2m_{\mathrm{A}}} + \frac{\hat{p}_{\mathrm{B}}^2}{2m_{\mathrm{B}}} + V(\hat{x}_{\mathrm{A}})$, but with general potential $V(\hat{x}_{\mathrm{A}})$. It is known that, when the potential changes slowly over the size of a wave packet, the wave packet moves approximately like a classical particle in the potential evaluated at the localization of the packet [6]. We can derive this statement from a Taylor expansion of the potential around position x_0:

$$V(\hat{x}_{\mathrm{A}}) = V(x_0) + \frac{dV(x_0)}{dx_0}(\hat{x}_{\mathrm{A}} - x_0) + \frac{1}{2}\frac{d^2V(x_0)}{dx_0^2}(\hat{x}_{\mathrm{A}} - x_0)^2 + \dots . \tag{10}$$

Hence,

$$\frac{dV(\hat{x}_{\mathrm{A}})}{d\hat{x}_{\mathrm{A}}} = \frac{dV(x_0)}{dx_0} + \frac{d^2V(x_0)}{dx_0^2}(\hat{x}_{\mathrm{A}} - x_0) + \frac{1}{2}\frac{d^3V}{dx_0^3}(\hat{x}_{\mathrm{A}} - x_0)^2 + \dots , \tag{11}$$

and similarly for higher derivatives. We can now take the expansions (11) around the mean value x_0 and apply it to a state centered in position x_0, that is sufficently localised such that all the terms of order higher than zero in (11) have negligible norm. When we apply the operator (11) to this state, we obtain $\frac{dV(\hat{x}_{\mathrm{A}})}{d\hat{x}_{\mathrm{A}}} |\psi\rangle \approx \left.\frac{dV(\hat{x}_{\mathrm{A}})}{d\hat{x}_{\mathrm{A}}}\right|_{x_0} |\psi\rangle$. We assume that the same approximation is also valid for the time-evolved state of A after an infinitesimal time interval δt. Under this approximation the quantum state of A evolves, in the time δt, as if it were constantly accelerated. If we now consider a superposition of coherent states, localised around two positions x_1 and x_2, the whole system evolves as if it were in a superposition of accelerations.

For a general potential, in order to calculate the displacement of the new reference frame A, $\hat{X}_{\mathrm{A}}(t) = \hat{x}_{\mathrm{A}}(t) - \hat{x}_{\mathrm{A}}$, we use the Trotter approximation, which at the second order in δt reads $e^{(A+B)\delta t} \approx e^{A\frac{\delta t}{2}} e^{B\delta t} e^{A\frac{\delta t}{2}}$, and gives the correct expression in the limit $\delta t \to 0$. This leads to

$$\hat{X}_{\mathrm{A}}(\delta t) = \frac{\hat{p}_{\mathrm{A}}}{m_{\mathrm{A}}}\delta t - \frac{1}{2}\frac{1}{m_{\mathrm{A}}}\frac{dV(\hat{x}_{\mathrm{A}})}{d\hat{x}_{\mathrm{A}}}\delta t^2 + O(\delta t^3). \tag{12}$$

To the first order in δt, this leads to redefine the $\hat{Q}_{\delta t}$ operator in Methods-The weak equivalence principle in quantum reference frames

$$\hat{Q}_{\delta t} = e^{-\frac{\mathrm{i}}{\hbar}\frac{m_{\mathrm{B}}}{m_{\mathrm{A}}}\left(\hat{p}_{\mathrm{A}} - \frac{dV(\hat{x}_{\mathrm{A}})}{d\hat{x}_{\mathrm{A}}}\delta t\right)\hat{x}_{\mathrm{B}}} e^{\frac{\mathrm{i}}{\hbar}\frac{\hat{p}_{\mathrm{A}}\hat{p}_{\mathrm{B}}}{m_{\mathrm{A}}}\delta t} e^{-\frac{\mathrm{i}}{\hbar}\frac{m_{\mathrm{B}}}{m_{\mathrm{A}}}\frac{\hat{p}_{\mathrm{A}}^2}{2m_{\mathrm{A}}}\delta t}, \tag{13}$$

where now $\frac{dV(\hat{x}_{\mathrm{A}})}{d\hat{x}_{\mathrm{A}}}$ does not commute anymore with $\hat{p}_{\mathrm{A}}$. As in the main text, we may apply this transformation to coherent states of A with both well localized position and momentum and superpositions thereof.

The Hamiltonian in the frame of A is then, to the lowest order in δt,

$$\hat{H}_{\mathrm{BC}}^{(\mathrm{A})} = \frac{\hat{\pi}_{\mathrm{B}}^2}{2m_{\mathrm{B}}} + \frac{\hat{\pi}_{\mathrm{C}}^2}{2m_{\mathrm{C}}} + \frac{m_{\mathrm{C}}}{m_{\mathrm{A}}} V(-\hat{q}_{\mathrm{C}}) - \frac{m_{\mathrm{B}}}{m_{\mathrm{A}}} \frac{dV}{dx_{\mathrm{A}}}\Big|_{-\frac{m_{\mathrm{C}}}{m_{\mathrm{A}}}\hat{q}_{\mathrm{C}}} \hat{q}_{\mathrm{B}} + \frac{1}{2}\left(\frac{m_{\mathrm{B}}}{m_{\mathrm{A}}}\right)^2 \frac{d^2V}{dx_{\mathrm{A}}^2}\Big|_{-\frac{m_{\mathrm{C}}}{m_{\mathrm{A}}}\hat{q}_{\mathrm{C}}} \hat{q}_{\mathrm{B}}^2 + O\left(R\right), \tag{14}$$

where $R = \left\{\delta t, \frac{d^3V}{dx_{\mathrm{A}}^3}\Big|_{-\frac{m_{\mathrm{C}}}{m_{\mathrm{A}}}\hat{q}_{\mathrm{C}}}\right\}$. When this Hamiltonian is applied to the state transformed to the reference frame of A we can neglect the higher derivatives of the potential. This means that the same conclusions as in Methods-The weak equivalence principle in quantum reference frames hold, i.e. the weak equivalence principle is generalised, for an interval of time δt, to when the reference frame is a quantum particle in superposition of accelerations.

In particular, when the potential $V(\hat{x}_{\mathrm{A}})$ is a Newtonian gravitational potential, it is possible to find a limit in which a quantum particle A in a gravitational field moves, for a time δt, as if it were in a superposition of uniform gravitational fields. The generalisation of the weak equivalence principle presented in this section then allows us to conclude that this constitutes a local frame which is equivalent to a frame moving in a superposition of accelerations, thereby extending the results known in the standard treatment of reference frames.

SUPPLEMENTARY REFERENCES

[1] G. Rosen, Galilean Invariance and the General Covariance of Nonrelativistic Laws, Am. J. Phys. **40.5**, 683-687 (1972).

[2] D. M. Greenberger, Inadequacy of the Usual Galilean Transformation in Quantum Mechanics, Phys. Rev. Lett. **87.10**, 100405 (2001).

[3] D. M. Greenberger, Some remarks on the extended Galilean transformation, Am. J. Phys. **47.1** 35-38 (1979).

[4] Will, Clifford M. "The confrontation between general relativity and experiment." Living Reviews in Relativity **9.1**, 3 (2006).

[5] G. Parravicini, Schrödinger Hamiltonians as affine connections, Il Nuovo Cimento A (1965-1970) **103.10**, 1403-1416 (1990).

[6] A. Peres, *Quantum theory: concepts and methods*, Vol. 57. Springer Science and Business Media, 2006.
